# Examining gender and sexual orientation differences in physical intimate partner violence experienced and perpetrated by youth living in eThekwini district South Africa during the COVID-19 pandemic

**DOI:** 10.1186/s12889-023-17199-x

**Published:** 2023-11-21

**Authors:** Kalysha Closson, Bongiwe Zulu, Julie Jesson, Janan J. Dietrich, Tatiana Pakhomova, C. Andrew Basham, Mags Beksinska, Angela Kaida

**Affiliations:** 1 Faculty of Health Sciences, Simon Fraser University, Burnaby, Canada; 2 Center on Gender Equity and Health, School of Medicine, University of California, San Diego, California USA; 3 Maternal Adolescent and Child Health Research Unit (MRU), Faculty of Health Sciences, University of the Witwatersrand, Durban, South Africa; 4 Center for Epidemiology and Research in POpulation Health (CERPOP), Université de Toulouse, Toulouse, France; 5 Perinatal HIV Research Unit (PHRU), Faculty of Health Sciences, University of the Witwatersrand, Johannesburg, South Africa; 6 Health Systems Research Unit, South African Medical Research Council, Cape Town, South Africa; 7 Division of Pharmacoepidemiology and Pharmacoeconomics, Brigham and Women’s Hospital/Harvard Medical School, Boston, MA USA

**Keywords:** Youth, South Africa, Intimate partner violence, LGBTQ+, COVID-19, Gender

## Abstract

**Background:**

Young women and Lesbian, Gay, Bisexual, Trans, Non-binary/no gender, or Questioning (LGBTQ+) youth in South Africa face some of the highest global levels of intimate partner violence (IPV). Given limited evidence in the wake of the COVID-19 pandemic, which has fuelled IPV globally, we aimed to describe and compare experiences and perpetration of IPV of youth aged 16–24 by sexual orientation and gender identity (SOGI).

**Methods:**

During the study period (December 2021-May 2022), youth aged 16–24 from eThekwini district, South Africa completed an online survey to understand multilevel impacts of the pandemic on youth. Participants were asked about experiences and perpetration of physical IPV since the start of the COVID-19 pandemic (March 2020). Descriptive statistics and adjusted logistic regressions compared the likelihood of experiencing and/or perpetrating physical IPV between cisgender and transgender inclusive heterosexual men; heterosexual women; gay, bisexual, or questioning men [GBQM]; lesbian, gay, bisexual, or questioning women [LGBQW]; or gender/sexual non-conforming youth [non-conforming].

**Results:**

Of 1,588 youth (mean age = 21.7 [SD = 2.3]; 71.7% Black) with non-missing SOGI and physical IPV data, 238 (15.0%) were LGBTQ+ (40.3% LGBQW and 36.1% non-conforming). Overall, 14.6% of respondents experienced physical IPV and 9.8% perpetrated physical IPV since the start of the pandemic, which differed by SOGI (12.3% of heterosexual men, 13.9% of heterosexual women, 22.0% of GBQM, 18.2% of LGBQW, and 25.0% of non-conforming youth experienced and 10.3% of heterosexual men; 7.7% of heterosexual women; 10.0% of GBQM; 18.2% of LGBQW; and 16.7% of non-conforming youth perpetrated). In adjusted models, compared to heterosexual women, non-conforming youth had increased odds of experiencing (adjusted odds ratio [aOR] = 2.36; 95%CI, 1.26–4.39) physical IPV and compared to heterosexual men, non-conforming youth had greater odds of perpetrating physical IPV (aOR = 2.19; 95%CI, 1.07–4.48) during the pandemic.

**Conclusion:**

Over one in six youth in our study experienced and one in ten perpetrated physical IPV since the onset of the COVID-19 pandemic, with gender and sexual non-conforming youth experiencing and perpetrating IPV at significantly greater rates than cisgender/heterosexual peers. Our findings highlight the need for gender transformative efforts that move beyond the gender binary to support healthy relationships and IPV prevention for LGBTQ + youth in South Africa and globally.

## Introduction

Over one in four women will experience some form of intimate partner violence (IPV) in their lifetime [1]. Experiences of IPV begin at an early age, with past-year IPV being higher among women aged 15–24 years than any other age group [1]. While global estimates of IPV perpetration by men are limited, rates in select populations including military populations (26%) [2] and youth in disadvantaged communities in Johannesburg, South Africa (40%) are similarly high or higher than global estimates of IPV experiences [3]. The vast majority of studies have focused on experiences and perpetration of IPV in heterosexual relationships. However, IPV perpetration by women as well as perpetration and experiences within non-heterosexual relationships have also been reported [4]. A recent review reported that IPV experiences and perpetration within non-heterosexual relationships happen at rates comparable to heterosexual relationships [5]. However, studies involving non-heterosexual participants have primarily explored IPV experiences within the relationships of gay and bisexual men, leaving gaps in the literature and understanding about IPV among LGBTQ + communities [6–8].

Across the globe, there is growing evidence that the COVID-19 pandemic and the subsequent public health response to “stay home” through legally enforced and self-imposed social distancing, fueled environments in which IPV could thrive [9]. Evidence suggests that LGBTQ + communities may have faced additional challenges with mental health and experiences of discrimination and violence, including IPV amidst the global lockdown [10–13]. Among LGBTQ + and non-LGBTQ + communities, situational stressors related to the COVID-19 pandemic, such as economic stress, social isolation, and pandemic-related stress have been associated with increased IPV experiences and perpetration [14–16]. As the onset of IPV often begins in adolescence and early adulthood, and in turn increases the risk of violence victimization and perpetration into adulthood, it is critical that research efforts focus on mitigating experiences and perpetration of IPV in this population [17–20]. Thus, while recent research is highlighting the added risk and experiences of IPV during the pandemic, the experiences of youth, the majority of whom are or were non-cohabitating during the pandemic, are less understood [21, 22].

In South Africa, rates of IPV were at critical levels pre-pandemic, with 21% of women experiencing physical IPV in their life-times [23]. Within sub-Saharan African settings of high sustained IPV, including South Africa, much research has explored the drivers and factors associated with IPV experiences and perpetration. Education, employment, and having children are drivers of IPV experiences [24, 25]; while alcohol use [26], experiences of violence during childhood or witnessing violence in childhood being significant drivers of IPV perpetration [24]. Early reporting from the South African Police Service (SAPS) suggested that reports of IPV in South Africa actually decreased by up to 68% from prior pandemic reporting periods [27, 28] yet domestic abuse hotlines utilization was reported to have increased by 65–100% during the initial lockdown [29]. IPV reporting has been made especially challenging and unreliable during periods of lockdown in which victims of IPV were confined at home with their perpetrator, having little to no support services, and no ability to report violence to the police [30, 31].

These data have begun to shine a light on the experiences and perpetration of IPV within South Africa during the pandemic, however, certain populations, including LGBTQ+ youth, have been left out of the conversation. While South Africa has progressive laws preventing LGBTQ + discrimination and violence and was the first African nation to make homosexuality and gay marriage legal (since 1998 and 2004, respectively), there exist vast disparities between the law and the everyday lived experiences of LGBTQ + individuals in South Africa [32, 33]. Moreover, the historical invisibility of IPV within LGBTQ + communities [34, 35] likely contributes to a lack of knowledge and awareness of IPV risks and support options [5], which may be particularly salient for LGBTQ + youth, many of whom are entering their first intimate relationship. Thus, while research around the globe has highlighted the exacerbating effects of the pandemic on existing health inequities, including increased systemic and structural violence towards LGBTQ + communities and youth [36, 37], it is imperative to characterize and compare experiences of perpetration of IPV during the pandemic across sexual orientation and gender identity (SOGI).

The aim of this study is to describe and compare the experiences and perpetration of physical intimate partner violence by SOGI among youth aged 16–24 living in eThekwini district, KwaZulu Natal, South Africa during the COVID-19 pandemic.

## Methods

### Study setting

AYAZAZI RIGHTS (Rapid Investigation of Gendered Health outcomes in the Time of Sars-Cov-2) took place over six months between December 2021 and May 2022, recruiting youth aged 16–24 from across eThekwini district South Africa. AYAZAZI stems from prior South African adolescent and youth health research [38–41], where AYA stands for adolescents and young adults, and “Zazi” meaning knowing themselves in isiZulu. The city of Durban, situated within eThekwini district, has been criticized for conservative views on LGBTQ + acceptance [42] and the lack of political response to the high sustained incidents of LGBTQ + hate crimes occurring within and around the city in recent years [43].

### Study Design

AYAZAZI RIGHTS was a cross-sectional, on-line health survey which aimed to explore youth experiences of the COVID-19 public health response and impacts on sexual, reproductive, and mental health outcomes. Youth aged 16–24 years, living in the eThekwini district, Durban, South Africa, who could read in English and/or isiZulu and had access to a mobile phone, tablet, or computer that could access the internet were eligible for participation. This study includes all participants who had complete data on gender, sexual orientation, experiences of IPV and all measured confounding factors. To improve accessibility, the survey was delivered to participants via the data-free Moya messenger app which allowed participants to complete the survey without using data from their mobile devices.

### Recruitment

A multi-pronged recruitment strategy was used to enrol participants. Recruitment strategies included contacting and inviting eligible former participants from studies led by WitsMRU who have agreed to be recontacted for other research projects; through the Wits MRU; community-based and youth-led organisations; and Community Advisory Boards (CABs), especially the Adolescent CAB (ACAB) which includes girls from High schools and Tertiary institutions from in and around the Durban area, as well as Senior CAB members of the MRU. Study information was also distributed via flyers in areas highly frequented by youth such as commercial retail settings, transit areas on their way into work or school, and CAB offices. The survey was also advertised online and via e-mails to various local institutions including on the WitsMRU website and social media platforms of multiple community organizations. Participants also had the opportunity to share the link and the QR code of the survey to their eligible peers and family members.

To promote the study widely, participants who completed the full mobile survey were eligible to enter a cash prize draw of R100 (CAD$ 8.50). The chances of winning ranged from one in every five to one in every twenty over the enrolment period.

### Ethical considerations

Ethical approval was provided by the Simon Fraser University Research Ethics Board and the UBC Behavioural Research Ethics Board (REB number: H21-02027), and by the University of the Witwatersrand Human Research Ethics Committee (Wits HREC-Medical) in South Africa (REB number: M210863). Participants were provided with an electronic informed consent letter, detailing the purpose of the survey, benefits and risk for participation, and key contacts for further question prior to accessing to the questionnaire. Informed consent was obtained from all participants prior to completing the online survey. A list of resources including online and in-person local support services such as for mental health and sexual and reproductive health care was provided at the end of the questionnaire.

### Data collection

The questionnaire was auto-administered and developed in English and translated in isiZulu, with back translation into English to ensure accuracy. They survey was piloted with community members, research assistants and various experts prior to being launched. Overall, the median time of survey completion was 15 min (Interquartile Range [IQR] = 8–26). Participants who completed the whole survey in less than 4.5 min were subsequently excluded from the analyses as our team concluded that any amount of time under 4.5 min would be insufficient to fully read, understand, and complete the full survey. Other quality assessments were made to identify potential outliers and duplicates, such as screening for very similar final comments, exact same responses in the demographics’ section of the survey, as well as same phone number. Those steps did not lead us to further exclude participants. The mobile health survey and associated database were created using REDCap (Research Electronic Data Capture) [44].

### Outcome variables

Physical IPV experiences were captured by asking respondents “Since the start of the COVID-19 pandemic (March 2020) (or since the start of your relationships if after the pandemic began) has your partner slapped you, hit you, kicked you, thrown things at you, or done anything else to physically hurt you?” (yes vs. no). While physical IPV perpetration was captured by asking respondents “Since the start of the COVID-19 pandemic (March 2020) (or since the start of your relationships if after the pandemic began) have you slapped, hit, kicked, thrown things at, or done anything else to physically hurt your partner?” (yes vs. no). These measures were similar to other youth-based South African IPV studies [45, 46].

#### Other IPV-related variables of interest

For each of the IPV questions, participants were also asked did this occur more often or less often since the COVID-19 pandemic (more often; same; less often).

### Exposure of interest

The exposure of interest in this study was SOGI. This was measured by asking participants what is your gender identity ((1) boy/man; (2) girl/woman; (3) non-binary; (4) I do not identify with any specific gender; (5) Prefer to self-identify; (6) Prefer not to say) and what is your sexual orientation? ((1) heterosexual/straight; (2) lesbian or gay; (3) bisexual; (4) I do not identify with any specific sexual orientation; (5) undecided/questioning; (6) prefer not to say; or (7) Other, please specify: _____). We combined the above categories to explore SOGI differences in the risk of IPV between cisgender and transgender inclusive heterosexual women; heterosexual men; lesbian, gay, bisexual or questioning women (LGBQW), gay, bisexual, or questioning men (GBQM), and any youth identifying as non-binary or that selected that they did not identify with any gender or sexual orientation were categorized as non-conforming.

### Potential confounders

We adjusted for potential confounding by age (continuous), race (Black; Indian or Asian or white [ref]; Coloured [in the South African context, Coloured is a heterogenous racial group consistent of individuals of mixed racial heritage]), school/employment status (currently in school or employed vs. not in school or employed), relationship status (not in a current relationship [ref]; in a relationship, living together; in a relationship not living together), any children (yes vs. no), alcohol use frequency during the pandemic (once or twice per week or more; less than monthly or once per month; never), experiencing economic hardship during the pandemic, and increased stress. Economic hardship was assessed by combining two measures of increased difficulties in accessing food or decreased income since the onset of the pandemic. Participants indicating that they had a harder time accessing food and/or that their income decreased were coded as having increased hardship during the pandemic. Increased mental stress was measured by first asking participants if they felt 1) anxious, scared, 2) worried, 3) upset, angry, and 4) as if they were not coping and for each of the four questions, if they responded yes were then asked, ‘Was this different than before the COVID-19 pandemic?” (more than usual, less than usual, or the Same). Participants who responded yes and then more than usual to any of the four mental health variables were coded as having increased stress during the pandemic.

### Statistical analyses

Characteristics of the study population were described overall and by SOGI using frequency (%) for categorical variables and means (SD) for numeric variables. The Standard Mean Difference (SMD) was calculated to show imbalance between demographic variables and SOGI. SMD was selected over p-values as it not influenced by sample size and allows for comparison of balance across variables measuring different units [47, 48]. SMDs of 0.2, 0.5, and 0.8 represent small, medium, and large effect sizes, respectively [48, 49]. Bivariate associations between SOGI and other socio-demographic characteristics with experiences and perpetration of physical IPV during the pandemic were evaluated using unadjusted odds ratios. Multivariable logistic regressions estimated the association between SOGI and (a) experiencing, and (b) perpetrating physical IPV, which was expressed though crude and adjusted odds ratios (ORs) and 95% confidence intervals (95% CI) to provide a measure of precision for the ORs. Heterosexual women were selected as the reference group for the IPV experience model and heterosexual men as the reference group for the IPV perpetration model as they have been the demographic group of interest in IPV experience and perpetration research, respectively. The multivariable logistic regression models were adjusted for age, race, sex, current school/employment status, relationship status, any children, and alcohol use frequency during the pandemic. We considered ORs with 95% CIs that did not include 1.00 to be statistically significant at the α = 0.05 level. However, in considering public health significance, the magnitude of association was of primary interest in our interpretations, rather than statistical significance.

## Results

**Fig. 1:**
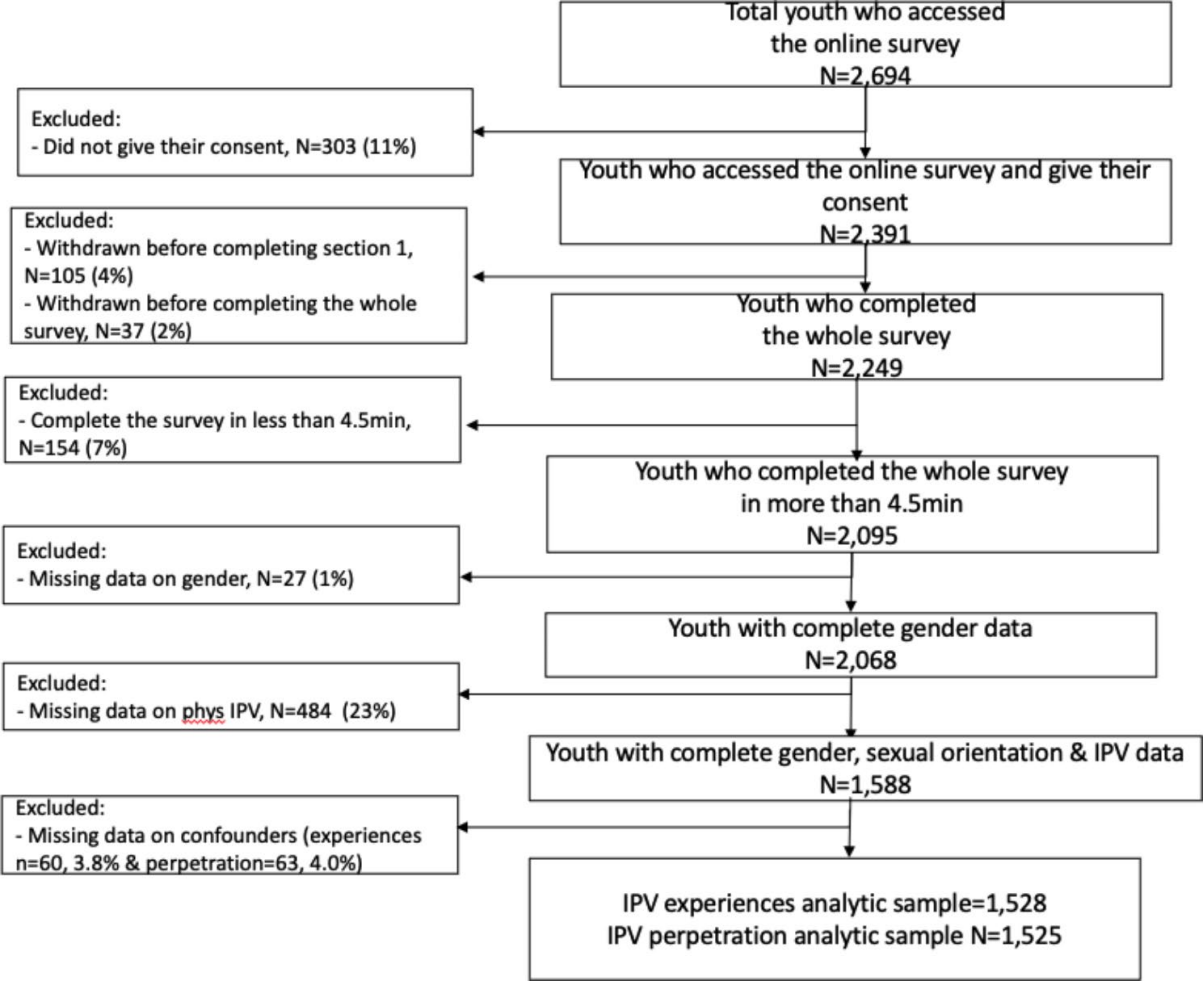
Flowchart of analytic sample of youth aged 16–24 living in eThekwini District, South Africa included in this study

Overall, 2694 youth were reached and accessed the online survey. Among them, 303 (11%) did not consent to participate, 142 (6%) participants did not complete the entire survey. Among the 2,249 participants that completed the entire survey, we excluded 154 (7%) who completed it in less than 4.5 min, leading to a final sample of 2,095. For this analysis, we restricted our sample to participants who had available data on gender, sexual orientation, and IPV leading to a final analytic sample of 1,588, with 1,528 and 1,525 participants with non-missing potential confounding variable data included in the adjusted IPV experiences and perpetration analyses, respectively (see flowchart in Fig. 1 for study selection).

### Sample characteristics

Of 1,588 youth (47.7% heterosexual women and 14.9% lesbian, gay, bisexual, questioning, or gender and/or sexual non-conforming youth, mean age = 21.7 [SD = 2.34]) who had valid gender and physical IPV data, 3.5% were GBQM, 6.0% were LGBQW, and 5.4% were non-conforming. The majority of respondents were Black (73.0%), in school or currently employed (69.4%), and in a current relationship not living with their partner (77.1%). Compared to the overall sample, a larger proportion of non-conforming youth were Black (88.4%), in a current relationship, not living with their partner (92.9%), reported drinking once or twice per week or more during the pandemic (55.8%), and a smaller proportion of non-conforming (< 5–10%) had children (Table 1).

**Table 1:** Sample characteristics by sexual orientation and gender identity of youth aged 16–24 living in eThekwini District of South Africa during the COVID-19 pandemic (n = 1588)

	Overall n (%)	Heterosexual women n (%) 758 (47.7)	Heterosexual man n (%) 592 (37.3)	Gay, bisexual, or questioning men n (GBQM) (%) 56 (3.5)	Lesbian, gay, bisexual or questioning woman (LGBQW) n (%) 96 (6.0)	Gender and Sexual Non-conforming youth (Non-conforming) n (%) 86 (5.4)	SMD*
Characteristics							
**Age mean, SD**	21.73 (2.34)	21.74 (2.45)	21.78 (2.31)	21.09 (2.34)	21.28 (2.07)	22.17 (1.63)	0.253
**Race/Ethnicity**							
Black	1152 (73.0)	530 (70.0)	438 (74.2)	44 (80.0)	68 (70.8)	76 (88.4)	0.322
Coloured	181 (11.5)	86 (11.4)	62 (10.5)	<10 (10–15%)	20 (20.8)	<10 (5–10%)	
white, Indian, or Asian	246 (15.6)	141 (18.6)	90 (15.3)	<10 (5–10%)	8 (8.3)	<5 (1–5%)	
Unknown	4						
**Employed and/or in school**: Yes	1093 (69.4)	489 (64.9)	426 (73.2)	43 (76.8)	72 (75.0)	63 (73.3)	0.114
Unknown	14						
**Any economic hardship during the pandemic: Yes**	817 (52.2)	403 (53.6)	277 (47.4)	37 (74.0)	56 (60.2)	44 (51.8)	0.258
Unknown	24						
**Relationship Status**							0.497
No relationship	210 (13.3)	96 (12.7)	69 (11.8)	19 (33.9)	22 (23.2)	<5 (1–5%)	
In relationship, living together	151 (9.6)	88 (11.6)	40 (6.8)	<10 (5–10%)	14 (14.7)	<5 (1–5%)	
In relationship not living together	1218 (77.1)	572 (75.7)	478 (81.4)	30 (53.6)	59 (62.1)	79 (92.9)	
Unknown	9						
**Any Children**: Yes	682 (43.1)	382 (50.5)	243 (41.3)	15 (26.8)	37 (38.5)	<10 (5–10%)	0.507
Unknown	6						
**Alcohol use during the pandemic**							0.527
once or twice per week or more	417 (26.5)	111 (14.7)	209 (35.5)	22 (39.3)	27 (28.4)	48 (55.8)	
less than monthly or once a month	524 (33.2)	289 (38.3)	173 (29.4)	10 (17.9)	26 (27.1)	26 (30.2)	
never	639 (40.4)	355 (47.0)	206 (35.0)	24 (42.9)	42 (44.2)	12 (14.0)	
Unknown	8						
**Stress levels during the pandemic**							0.206
More stressed	865 (54.8)	477 (63.3)	248 (42.0)	30 (53.6)	57 (61.3)	53 (61.6)	
Not more stressed	714 (45.2)	277 (36.7)	342 (58.0)	26 (46.4)	36 (38.7)	33 (38.4)	
Unknown	9						
**Experience of Physical IPV change since before the pandemic (n = 228)^**							
Same	116 (50.9)	57 (57.0)	37 (49.3)	8 (66.7)	<10 (30–35%)	<10 (35–40%)	0.529
Less	99 (43.4)	39 (39.0)	34 (45.3)	<5 (15–20%)	11 (57.9)	13 (59.1)	
More	13 (5.7)	<5 (1–5%)	<5 (5–10%)	<5 (15–20%)	<5 (10–15%)	<5 (5–10%)	

^Only among 228 participants who experienced physical IPV and who reported change in IPV experience since before the COVID-19 pandemic

Proportions for small cell sizes < 5 or < 10 were provided as a range to protect anonymity

IPV, intimate partner violence; SD, Standard Deviation; SMD, Standard Mean Difference

*SMDs of 0.2, 0.5, and 0.8 represent small, medium, and large effect sizes

**Fig. 2:**
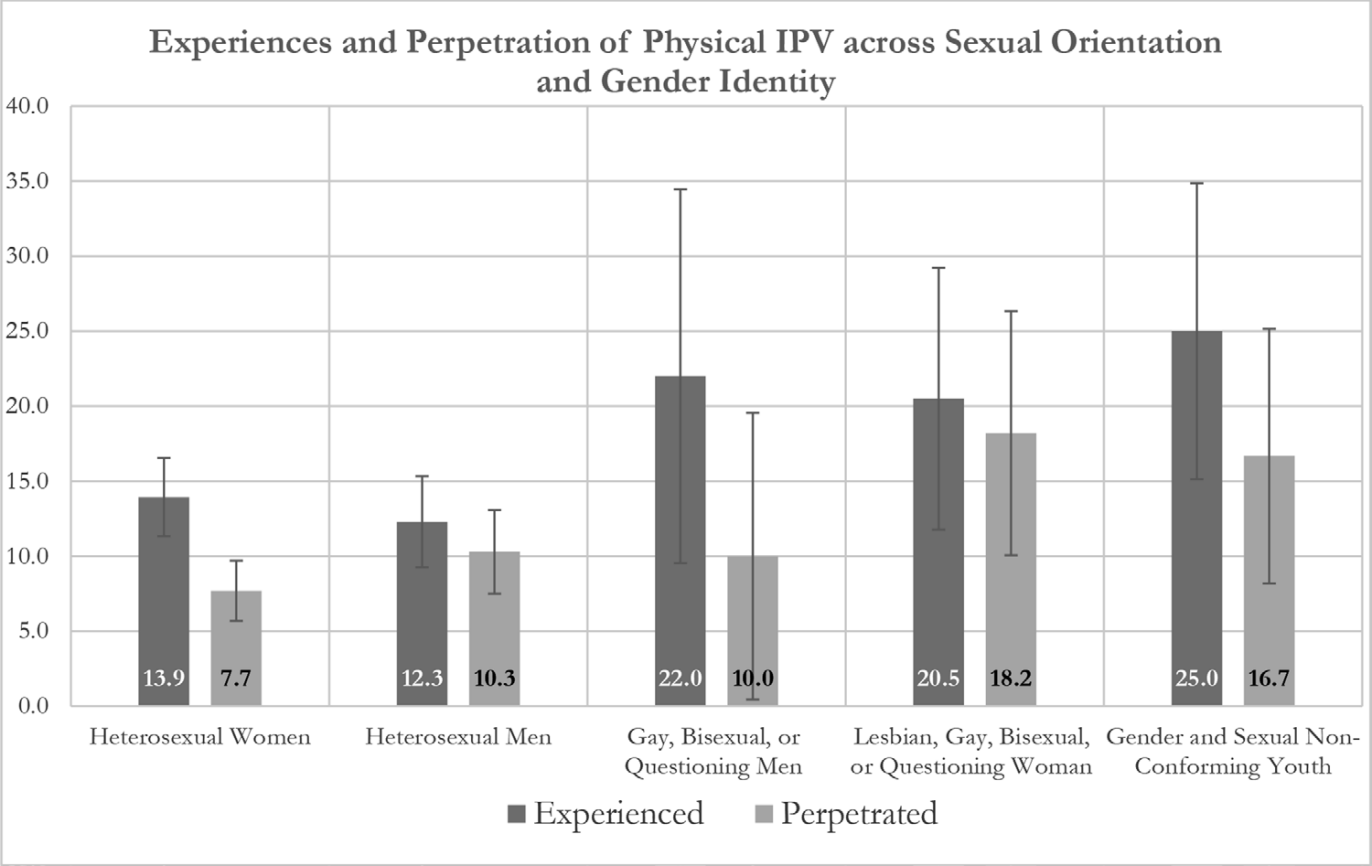
Proportion of youth living in eThekwini District of South Africa who experienced (left) or perpetrated (right) physical intimate partner violence over the course of the COVID − 19 pandemic (March 2020 to interview date [January to May 2022] or since their relationship started if after March 2020

Overall, 14.6% of respondents experienced physical IPV, 9.8% perpetrated physical IPV, and 6.5% reported both perpetrating and experiencing physical IPV at some point between the start of the COVID-19 pandemic and their interview date, with 25.0% of non-conforming youth, 22.0% of GBQM, 20.5% of LGBQW, 12.3% of heterosexual men, and 13.9% of heterosexual women reporting experiencing physical IPV during the pandemic and 18.2% of LGBQW, 16.7% of non-conforming youth, 10.0% of GBQM, 10.3% of heterosexual men, and 7.7% of heterosexual women reporting perpetrating physical IPV during the pandemic (Fig. 2).

Of note, all GBQM and non-conforming youth who perpetrated physical IPV during the COVID-19 pandemic also experienced physical IPV during the COVID-19 pandemic. Among the 240 respondents reported experiencing physical IPV during the COVID-19 pandemic, 228 reported on whether the frequency changed during the pandemic, with 13 (5.7%) reported experiencing more physical IPV, 43.4% reporting less, and 50.9% reporting experiencing the same level of physical IPV during the COVID-19 pandemic. Sparse data limited our ability to explore differences in the change of IPV frequency during the pandemic by SOGI (Table 1).

At the bivariate level (Table 2), older age, Coloured (vs. Black) respondents, and those with children had greater odds of experiencing and perpetrating physical IPV, while white, Indian, and Asian respondents (vs. Black), those in a relationship, not living with their partner (vs. not in a relationship), and those who drank less than once a week had reduced odds of both experiencing and perpetrating physical IPV. Experiencing increased hardship during the pandemic and those living with their partner (vs. not in a relationship) also had greater odds of experiencing physical IPV during the pandemic.

**Table 2:** Bivariate differences of between youth aged 16–24 living in eThekwini District of South Africa who did and did not experience physical intimate partner violence (n = 1528) and who did and did not perpetrate physical intimate partner (n = 1525) violence during the COVID-19 pandemic

	Experienced physical IPV		Odds ratio	Perpetrated physical IPV		Odds ratio
	No 1305 (85.4)	Yes 223 (14.6)		No 1375 (90.2)	Yes 150 (9.8)	
**Exposure of interest: sexual orientation and gender identity**						
Heterosexual women	636 (86.1)	103 (13.9)	Ref	681 (92.3)	57 (7.7)	0.73 (0.50–1.07)
Heterosexual men	497 (87.7)	70 (12.3)	0.87 (0.63–1.20)	507 (89.7)	58 (10.3)	Ref
GBQM	39 (78.0)	11 (22.0)	1.74 (0.86–3.51)	45 (90.0)	5 (10.0)	0.97 (0.37–2.54)
LBQW	70 (81.8)	18 (18.2)	1.59 (0.91–2.77)	72 (83.3)	16 (18.2)	1.94 (1.06–3.56)
Non-conforming	63 (75.0)	21(25.0)	2.06 (1.20–3.52)	70 (5.1)	14 (16.7)	1.75 (0.93–3.30)
**Potential confounders**						
**Age mean, SD**	21.60 (2.42)	22.51 (1.64)	1.22 (1.13–1.31)	21.68 (2.40)	22.33 (1.64)	1.15 (1.05–1.24)
**Race/ethnicity**						
Black	955 (86.0)	155 (14.0)	Ref	1007 (90.9)	101 (9.1)	Ref
Coloured	127 (73.0)	47 (27.0)	2.28 (1.57–3.32)	135 (77.6)	39 (22.4)	2.88 (1.91–4.34)
white, Indian, or Asian	223 (91.4)	21 (8.6)	0.58 (0.36–0.94)	233 (95.9)	10 (4.1)	0.43 (0.22–0.83)
**Employed and/or in school**						
Yes	918 (86.5)	143 (13.6)	0.75 (0.56–1.02)	963 (90.9)	97 (9.2)	0.78 (0.55–1.12)
No	387 (82.9)	80 (17.1)	Ref	412 (88.6)	53 (11.4)	Ref
**Increased hardship during the pandemic**						
No	651 (88.8)	82 (11.2)	Ref	670 (91.7)	61 (8.3)	Ref
Yes	654 (82.2)	141 (17.7)	1.71 (1.28–2.29)	705(88.8)	89 (11.2)	1.39 (0.98–1.95)
**Relationship status**						
No relationship	154 (81.9)	34 (18.1)	Ref	158 (84.9)	28 (15.1)	Ref
In relationship, living together	99 (66.0)	51 (34.0)	2.33 (1.41–3.85)	115 (76.7)	35 (23.3)	1.72 (0.99–2.98)
In relationship not living together	1052 (88.4)	138 (11.6)	0.59 (0.39–0.90)	1102 (92.7)	87 (7.3)	0.45 (0.28–0.70)
**Any Children**						
Yes	530 (81.0)	124 (19.05)	1.83 (1.38–2.44)	568 (87.0)	85 (13.0)	1.86 (1.32–2.61)
No	775(88.7)	99(11.3)	Ref	807(92.5)	65(7.5)	Ref
**Alcohol use during the pandemic**						
once or twice per week or more	308 (77.29)	91 (22.8)	Ref	328 (82.4)	70 (17.6)	Ref
less than monthly or once a month	469 (90.5)	49 (9.5)	0.35 (0.24–0.51)	497 (95.9)	21 (4.1)	0.20 (0.12–0.33)
never	528 (86.4)	83 (13.6)	0.53 (0.38–0.74)	550 (90.3)	59 (9.7)	0.50 (0.35–0.73)
**Increased mental stress during the pandemic**						
Yes	709 (84.3)	132 (15.7)	Ref	763 (90.8)	77 (9.2)	Ref
No	596 (86.8)	91 (13.2)	0.82 (0.61–1.09)	612 (89.3)	73 (10.7)	1.18 (0.84–1.66)

Proportions for small cell sizes < 5 or < 10 were provided as a range to protect anonymity

GBQM, gay, bisexual, or questioning men; LBQW, lesbian, bisexual, or questioning women

In unadjusted analyses, compared to heterosexual women, non-conforming youth (OR = 2.06, 95%CI, 1.20–3.52) had greater odds of experiencing physical IPV during the pandemic (Table 2). After adjusting for potential confounders (Table 3), non-conforming youth remained at greater odds of experiencing physical IPV (vs. heterosexual women, aOR = 2.73; 95%CI, 1.47–5.06), and had greater odds of perpetrating physical IPV (vs. heterosexual men, aOR = 2.19; 95%CI, 1.07–4.48).

**Table 3:** Unadjusted and adjusted association between sexual orientation and gender identity and (1) Experiencing physical IPV (n = 1528) and (2) Perpetrating physical IPV (n = 1525)

	Experiencing IPV	Perpetrating IPV
	Adjusted Odds Ratio (95%CI)	Adjusted Odds Ratio (95%CI)
Heterosexual women	Ref	0.73 (0.48–1.13)
Heterosexual men	0.90 (0.63–1.30)	Ref
Gay, bisexual, or questioning men	1.50 (0.70–3.25)	0.75 (0.27–2.07)
Lesbian, gay, bisexual or questioning women	1.39 (0.75–2.57)	1.64 (0.83–3.23)
Gender non-conforming youth	**2.36 (1.26–4.39)**	**2.19 (1.07–4.48)**

Models adjusted for age, race/ethnicity, having any children, school/employment status, relationship status, economic hardship during the pandemic, mental health during the pandemic, and alcohol frequency during the pandemic

## Discussion

This study presents differences in experiences and perpetration of physical IPV faced by youth residing in eThekwini district of South Africa during the COVID-19 pandemic across gender and sexual orientation. Despite a large proportion of youth experiencing violence during the pandemic reporting that this was less than pre-pandemic times, our results indicated that among youth aged 16 to 24, over one in six experienced and one in ten perpetrated physical IPV during the pandemic. We found that gender and sexual non-conforming youth experienced 2.7 times the odds of physical IPV than heterosexual women. Novel findings from our study include two-times odds of non-conforming youth perpetrating IPV than heterosexual men. Across SOGI there was considerable overlap between physical IPV perpetration and experiences. Findings from our study corroborate global research demonstrating the high levels of experiences and perpetration of IPV within LGBTQ + relationships [50–52].

Given the focus and efforts put towards preventing heterosexual women from experiences of IPV, and supporting women who experience IPV, in South Africa [23, 53–56], our results demonstrating similar or lower levels of physical IPV experiences among heterosexual women were unexpected. Contrary to other studies conducted in South Africa highlighting the disproportionate levels of IPV experiences among woman compared to men [24], our study found that heterosexual young women and men experienced similar rates of physical IPV in their relationships. Our results do align with prior research among Kenyan adults [57] and youth in Tanzania [58] showing higher but similar levels of recent and lifetime IPV experiences and perpetration among women and men. High levels of both experiencing and perpetrating violence among men and women in heterosexual relationships has been previously reported among adults in Kenya, with a 2022 study showing that 23% of women and 18% of men experienced physical violence in their relationship in the 12 months prior to being interviewed [57]. The same study showed that IPV perpetration was higher among those also reporting experiencing IPV among both women and men [57]. Our data further adds to these findings that did not explore experiences and perpetration of IPV in non-heterosexual relationships, demonstrating that queer and gender non-conforming youth face and perpetrate IPV at higher levels than their heterosexual peers, apart from gay, bisexual, and questioning men who perpetrated the lowest levels of IPV in their relationships.

Our data aligns with global research highlighting elevated levels of IPV experiences within LGBTQ + relationships. For example, in the US CDC’s National Intimate Partner and Sexual Violence Survey (NIPSVS), 44% of lesbians and 61% of bisexual women have experienced rape, physical violence, or stalking by an intimate partner, compared to 35% of straight women [59]. Bisexual women had a significantly higher lifetime prevalence of rape, physical violence, and/or stalking by an intimate partner when compared to both lesbian and heterosexual women, while on the other hand lesbian women and gay men reported levels of IPV and sexual violence equal to or higher than those of heterosexuals [54]. A systematic review of 52 global studies of IPV within gay, bisexual, and other men who have sex with men found a pooled prevalence of physical IPV experiences at 17% and perpetration at 12% [60]. These global levels align with reporting among GBQM in our study of whom 25% experienced and 11% perpetrated physical IPV over the course of the pandemic. These data reveal a growing concern and documentation of experiences in the LGBTQ + community, and a need to focus IPV prevention and supports for LGBTQ + youth.

Levels of experiences and perpetration of physical IPV among youth aged 16–24 years in our study was higher than lifetime experiences of physical IPV reported by women of all ages in KwaZulu Natal (15.0% vs. 13.7%) and prior reports of physical IPV perpetration among young women aged 16–24 years across South Africa (10.0% vs. 4.6%) [23]. However, these data are difficult to compare as we explored experiences and perpetration across all genders and sexual orientations and our sample was limited to youth living in eThekwini District. In our study, 65% of youth reporting perpetrating physical IPV had also experienced physical IPV, with 100% of non-conforming youth and lesbian and bisexual young women who perpetrated physical IPV also having had experienced physical IPV. Higher perpetration among individuals who are survivors of violence is well documented in the literature globally and sub-Saharan Africa, which may be particularly prevalent in LGBTQ + relationships [61, 62]. For example, previous research among gay, bisexual, and other men who have sex with men in South Africa and Namibia found that 7.3% of men had experienced IPV from their partners and 10.2% reported experiencing bi-directional violence in the 12 months prior to being surveyed.

Multiple interacting factors are likely driving the elevated rates of IPV among LBGTQ + youth observed in our study. These may include individual-levels factors including internalized stigmas or homophobia, as well as minority stress, which might result in higher levels of violent behaviour towards in-group members, including one’s partner [63–65]. While South Africa has one of the most progressive laws and policies promoting LGBTQ + rights and protections [32], LGBTQ + discrimination across the country is still widespread and this includes discrimination and violence from intimate partner [66].

There is a dearth of research exploring the impact of the COVID-19 pandemic on the sexual and reproductive health outcomes inclusive of IPV of LGBTQ + youth [67], especially within low-and-middle income countries. While rates of IPV during the pandemic were high, reports of increased frequency during this period were extremely low, which is promising. Reduced rates may have been due to limited contact between partners during lockdown periods. Reduced experiences may have also been impacted by South Africa’s alcohol ban, which did result in reductions in violence and trauma unit admissions for alcohol-related injuries [68, 69]. Despite these reductions in experiences of physical IPV reported during the pandemic, our results signal a critical need for efforts to move beyond focusing on supporting and preventing IPV within heterosexual relationships to address high levels of experiences and perpetration faced by LGBTQ + youth in South Africa and globally. Efforts should include exploring how gender transformative efforts, such as SASA! and Stepping Stones Creating Futures, which have shown promise in reducing IPV experiences [70, 71] and perpetration [72] within heterosexual relationships in South Africa can be adapted to address IPV in LGBTQ + relationships. Gender transformative programming aims to move beyond individual-level efforts to address societal and community-level power and gender inequities to transform gender norms that perpetuate violence, especially IPV [73]. While these efforts have shown promise among women and men, by working to address inequitable gender norms and relations, there is limited understanding into how the success of these programs could expand to include individuals of all genders in non-heterosexual relationships. Current interventions aimed at preventing IPV are framed within heteronormative notions that women are passive victims of violence and men are perpetrators. Narrow conceptualizations of gender as binary, exclude individuals who do not conform to these narrow categories. Thus, leaving few to no options for support within these communities. This is concerning as our study found that non-conforming youth were both more likely to experience as well as perpetrate physical IPV. Efforts are needed to address experiences of IPV that are not solely focused on unidirectional IPV perpetrated by men towards women in heterosexual relationships. This should include efforts to better understand and seek to prevent and support bilateral IPV that might be occurring at higher rates among gender and sexual non-conforming and young queer women’s relationships.

### Strengths and limitations of this study

While much research has centered on youth, gender, and violence in the context of HIV in South Africa, most studies have been underpowered or lack data on experiences of LGBTQ + youth. We were able to explore experiences and perpetration of IPV across sexual orientation and gender identity. However, our results also present several limitations of note. To reduce missing data and questionnaire fatigue from the mobile health survey, we restricted the survey to take an average of 15 min to complete. This limited our ability to ask detailed questions about youth’s relationships and the context of the relationship and violence experienced and perpetrated. As such, findings demonstrating that physical IPV was reported as happening less than prior to the pandemic should be taken with caution. Also, as we only included a measure of current relationship status, some participants who were never in a relationship during the pandemic may have been including in our analysis, even if they were not at risk of experiencing of perpetrating IPV during the pandemic. Moreover, the study was cross-sectional, and the low reporting of changes to experiences of IPV before and after the pandemic limits our ability to make inferences about the extent to which individuals experiences were due to the COVID-19 pandemic, if these experiences were new, and relationship dynamics pre/post-COVID (e.g., length of relationship, gender and age of partner, if violence was being experienced/perpetrated by multiple or a single partner). We also did not ask about when during the pandemic the violence happened, which at the time of the survey was ~ 2 years since the public health emergency was declared, thus limiting comparability with other studies measuring IPV in the last 12 months or lifetime experiences. Missing data on IPV was likely influenced by respondents’ relationship experiences that were not captured through skip patterns in the survey due to the limited soape of the survey. We also did not explicitly ask about transgender identity which limits our ability to explore how experiences may have differed between transgender and cisgender youth. Finally, given the sensitive nature of these data the results may have been influenced by social desirability bias. However, our study design with data being collected completely anonymously via a mobile survey may have reduced this bias.

## Conclusion

Across gender identity and sexual orientation our results highlight that gender non-conforming youth report both experiencing and perpetrating IPV at significantly higher levels than their heterosexual peers, with over one-quarter of non-conforming youth having experienced physical IPV during the pandemic. During the first two years of the COVID-19 pandemic, young heterosexual women reported IPV levels similar to young men and lower IPV levels than queer men, queer women, and non-conforming youth. Our findings signal a critical need for research to better understand experiences and consequences of IPV within the relationships of LGBTQ + youth. Efforts need to move beyond the gender binary to address and create programming that works to prevent and support young people of diverse genders and sexual orientations in forming and experiencing healthy relationship dynamics free of violence in South Africa and globally.

## Data Availability

The de-identified data cannot be publicly shared as we do not have community or REB approval to do so. For researchers or trainees wishing to access the data, please contact Dr. Angela Kaida to request access.
